# Autophagy: A Multifaceted Partner in Liver Fibrosis

**DOI:** 10.1155/2014/869390

**Published:** 2014-08-31

**Authors:** Ariane Mallat, Jasper Lodder, Fatima Teixeira-Clerc, Richard Moreau, Patrice Codogno, Sophie Lotersztajn

**Affiliations:** ^1^INSERM U955, 94000 Créteil, France; ^2^Université Paris-Est, Faculté de Médecine, UMR-S955, 94000 Créteil, France; ^3^INSERM U1149, Center for Research on Inflammation, 75018 Paris, France; ^4^Université Paris Diderot, Sorbonne Paris Cité, Faculté de Médecine, Site Xavier Bichat, 75018 Paris, France; ^5^Laboratoire d'Excellence Inflamex, 75018 Paris, France; ^6^Département Hospitalo-Universitaire (DHU) UNITY, Service d'Hépatologie, Hôpital Beaujon, AP-HP, 92000 Clichy, France; ^7^INSERM U1151-CNRS UMR8223, INEM, 75014 Paris, France; ^8^Université Paris-Descartes, Sorbonne Paris Cité, Site Necker Enfants-Malades, 75015 Paris, France

## Abstract

Liver fibrosis is a common wound healing response to chronic liver injury of all causes, and its end-stage cirrhosis is responsible for high morbidity and mortality worldwide. Fibrosis results from prolonged parenchymal cell apoptosis and necrosis associated with an inflammatory reaction that leads to recruitment of immune cells, activation and accumulation of fibrogenic cells, and extracellular matrix accumulation. The fibrogenic process is driven by hepatic myofibroblasts, that mainly derive from hepatic stellate cells undergoing a transdifferentiation from a quiescent, lipid-rich into a fibrogenic myofibroblastic phenotype, in response to paracrine/autocrine signals produced by neighbouring inflammatory and parenchymal cells. Autophagy is an important regulator of liver homeostasis under physiological and pathological conditions. This review focuses on recent findings showing that autophagy is a novel, but complex, regulatory pathway in liver fibrosis, with profibrogenic effects relying on its direct contribution to the process of hepatic stellate cell activation, but with antifibrogenic properties via indirect hepatoprotective and anti-inflammatory properties. Therefore, cell-specific delivery of drugs that exploit autophagic pathways is a prerequisite to further consider autophagy as a potential target for antifibrotic therapy.

## 1. Liver Fibrosis

Liver fibrosis is defined by the excessive accumulation of extracellular matrix in response to chronic injury regardless of the cause. The condition arises from an altered wound-healing reaction designed in an attempt to reduce hepatic damage. Scar accumulation is the result of a bidirectional process combining increased synthesis and deposition of extracellular matrix proteins within the liver, and a parallel failure of physiological mechanisms underlying matrix turnover [1, 2]. Progression of fibrosis upon sustained liver insult is associated with expansion of fibrotic septa, ultimately leading to cirrhosis, which is a condition defined by fibrotic septa surrounding regenerating nodules and marked alterations of hepatic vascularisation. Whereas early stages of fibrosis do not generate any significant morbidity, cirrhosis carries a high risk of morbimortality, owing to severe complications of liver failure and portal hypertension (i.e., ascites, variceal bleeding, bacterial infections, hepatic encephalopathy, hepatorenal syndrome, acute-on-chronic liver failure, etc.) and to the high incidence of hepatocellular carcinoma in the cirrhotic liver [1, 2]. Given the high prevalence of several causes of liver diseases worldwide (e.g., alcohol, hepatitis B and C viruses, nonalcoholic fatty liver disease, etc.), cirrhosis is regarded as a high public health burden worldwide, representing the most common nonneoplastic cause of death among diseases of the gastrointestinal tract in Europe and the USA. Therefore, efficient antifibrotic therapeutic approaches are a high priority goal for hepatologists. In this respect, recent data have conclusively established, both in experimental models and in cohort studies, that eradication or efficient control of the cause of liver disease may be associated with regression of fibrosis and early stage cirrhosis [2]. However, this goal cannot be achieved in several instances, which justifies past and ongoing massive efforts to identify potential therapeutic antifibrotic targets.

## 2. Autophagy

Autophagy covers three catabolic processes (i.e., macroautophagy, microautophagy, and chaperone-mediated autophagy) responsible for the degradation of cell components in the lysosome [3, 4]. Macroautophagy (hereafter referred to as autophagy) is the most well characterized mechanism in eukaryotic cells and requires a vacuolar transport of cytoplasmic material to the lysosome. Autophagy starts with the formation of a double-membrane surrounded vacuole, known as the autophagosome, which ultimately fuses with the lysosomal compartment where autophagic cargoes are degraded. The autophagosome originates from the phagophore, a membrane that is nucleated and elongated by a family of autophagy-related (*ATG*) genes conserved between yeast and humans [5]. The phagophore formation is initiated by the UNC-51-like kinase 1 ULK1 (ATG1) complex in the omegasome, an endoplasmic reticulum (ER) based structure. The activity of this complex is controlled by the mammalian target of rapamycin complex 1 (mTORC1), which integrates diverse signals such as amino acids, glucose, and growth factors [6]. Upon mTOR inhibition by starvation, ULK1/2 dissociates from the complex and drives autophagosome formation, in a coordinated manner with the Beclin 1 (ATG6): vacuolar protein sorting 34 (Vps34, class III phosphatidylinositol 3-kinase) complex I. The synthesis of PtdIns3P by vacuolar protein sorting 34 (Vps34) is an important trigger for the elongation and closure of the autophagosome by two ubiquitin-like conjugation systems, ATG5-ATG12 and LC3 (ATG8)-PE (phosphatidylethanolamine).

Autophagy is an important regulator of liver homeostasis under physiological conditions [7–9]. The basal rate of autophagy is required to maintain liver homeostasis by elimination of aggregate-prone proteins and damaged mitochondria and by counteracting hepatocyte swelling [7–9]. The sequestration of mitochondria and protein aggregates mainly relies on the selective recognition of cargoes by autophagy adaptors, such as SQSTM1/p62, that bridge the cargoes to the autophagic machinery [3, 4]. SQSTM1/p62 contains a LC3-interacting region (LIR) that interacts with both LC3 and a UBA (ubiquitin-associated) domain, leading to the selective degradation of the ubiquitinated cargo by autophagy. SQSTM1/p62 also interacts with several signaling components, such as ERK1, *α*PKC, TRAF6, Keap1, and mTORC1 [3, 4]. Thus, regulation of cellular levels of SQSTM1/p62 by autophagy controls antioxidant defense, inflammatory response, cell growth, and apoptosis. Additional physiological functions of autophagy in the liver include regulation of metabolic pathways such as gluconeogenesis during fasting, *β*-oxidation of fatty acids, and ketone body formation. Amino acids used for gluconeogenesis are produced by proteolysis through bulk autophagy [10], whereas fatty acids are mainly produced by selective autophagy of triglycerides stored in lipid droplets (lipophagy) [11]. Autophagy probably also controls the level of very-low-density lipoprotein (VLDL) particles through lipophagy, which releases fatty acids and degrades apolipoprotein B. Moreover, liver autophagy plays a key role in restoring plasma glucose concentrations in neonates during fasting [12]. Finally, hepatocyte autophagy promotes liver regeneration after partial hepatectomy, by preserving the integrity of mitochondria and protecting hepatocytes from senescence [13].

Mounting evidence also indicates that alterations in the autophagic process in parenchymal and nonparenchymal liver cells drive or control the progression of various liver diseases, including alcoholic and nonalcoholic fatty liver disease, viral hepatitis, drug and ischemia-reperfusion injury, and hepatocellular carcinoma [7–9]. Novel findings also implicate autophagy in the control of liver fibrosis.

## 3. Cellular Effectors of Liver Fibrogenesis

### 3.1. Hepatic Myofibroblasts as Fibrogenic Cells of the Liver

**Figure 1:**
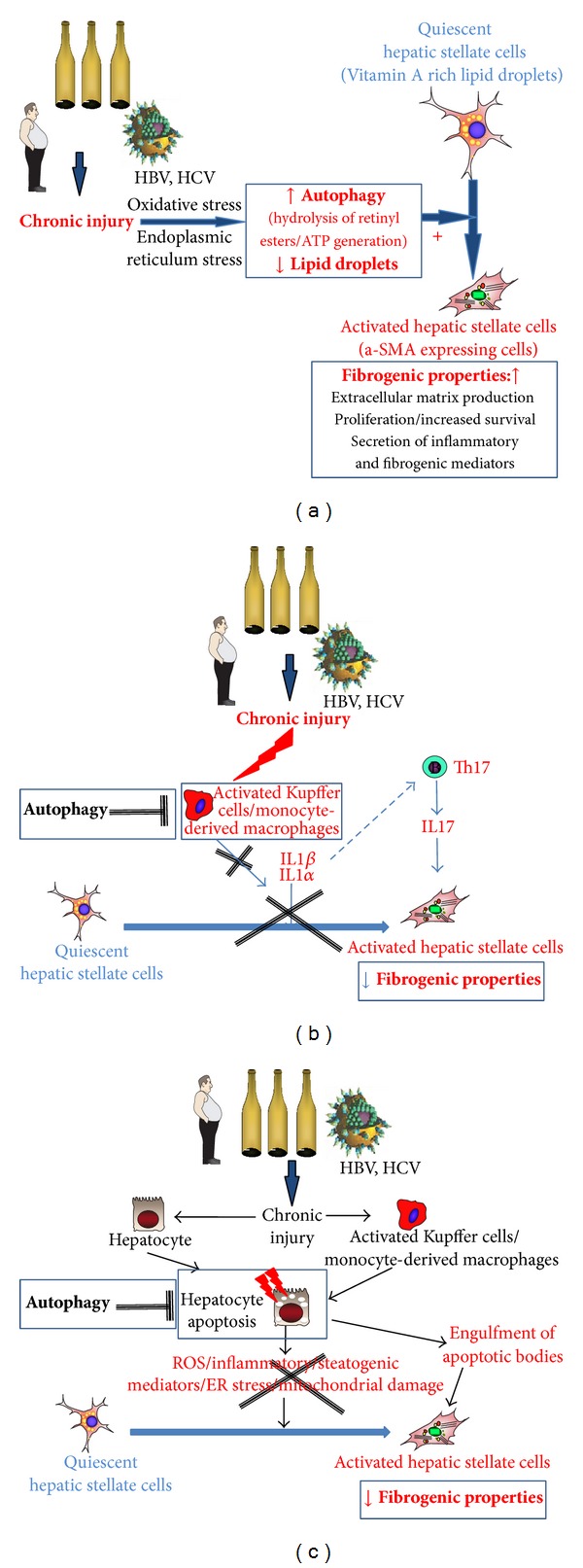
Impact of autophagy on the cellular effectors of liver fibrogenesis. (a) Autophagy drives hepatic stellate cell activation from a quiescent, lipid-rich to a myofibroblastic, fibrogenic phenotype. In response to chronic liver injury, quiescent hepatic stellate cells lose their retinyl ester-containing lipid droplets and acquire myofibroblastic features, associated with fibrogenic properties. Autophagy in hepatic stellate cells is stimulated by oxidative and endoplasmic reticulum stress and may serve to provide free fatty acids from retinyl esters, thereby supplying the energy required for initiating and perpetuating the fibrogenic phenotype. (b) Autophagy as a protective anti-inflammatory process with antifibrogenic properties. Hepatic macrophage autophagy stimulates an anti-inflammatory pathway, that reuses the production of IL1 alpha and IL1 beta, resulting in inhibition of liver fibrogenesis. In addition, inhibition of Th17 polarisation by IL1 alpha and beta may also contribute to the antifibrogenic effects of macrophagic autophagy. (c) Hepatoprotective properties of autophagy may contribute to inhibition of fibrogenesis. Autophagy is generally considered as a survival pathway for hepatocytes, therefore limiting oxidative stress profibrogenic pathways for hepatic stellate cells such as ER stress and mitochondrial damage.

Extracellular matrix accumulation during chronic liver injury is driven by a heterogenous population of myofibroblasts that migrate and accumulate at sites of liver injury in response to a wide variety of paracrine/autocrine signals produced by neighbouring inflammatory and parenchymal cells [1, 2]. Hepatic myofibroblasts display a fibrogenic phenotype (Figure 1(a)) characterized by (i) the secretion of an array of extracellular matrix proteins (ECM) predominating in fibrillar collagens, (ii) a high proliferative capacity and relative resistance to apoptosis, (iii) production of a wide range of ECM degrading enzymes (metalloproteinases, MMP) that modulate ECM remodeling and specific tissue inhibitors of the metalloproteinase family (TIMPs), and (iv) release of cytokines and growth factors that maintain a sustained inflammatory reaction and assist liver regeneration and angiogenesis. Several studies have shown that hepatic myofibroblasts of diverse origins coexist in the injured liver, with a large preponderance of cells derived from hepatic stellate cells and to a minor extent from resident portal fibroblasts [1, 2].

Hepatic stellate cells (HSC) represent the main source of liver fibrogenic cells. In the normal liver, HSC reside in the perisinusoidal space between endothelial cells and hepatocytes and display a quiescent phenotype characterized by the expression of a large panel of adipogenic genes and neural markers [1, 2]. A characteristic feature of quiescent HSC is the presence of cytoplasmic lipid vacuoles loaded with retinoids stored as retinyl esters and triglycerides. Upon acute or chronic liver injury, parenchymal injury and the resulting inflammatory reaction generate a large panel of signals that promote induction of specific sets of transcription factors and morphogens (Hedgehog ligands, Wnt) in quiescent HSC, thereby triggering the activation program and the acquisition of fibrogenic and proinflammatory properties [1, 2, 14–16]. Upon activation, quiescent HSC lose their retinyl ester-containing lipid droplets and the expression of adipogenic/lipogenic factors. In parallel, they acquire myofibroblastic-like features, including the expression of smooth muscle alpha actin, and* de novo* expression of receptors for fibrogenic, chemotactic, and mitogenic factors [1, 2, 14–16]. The activation process occurs in response to classical signals including lipid peroxides reactive oxygen species, proinflammatory and mitogenic cytokines and growth factors, and the matrix itself via integrin-mediated pathways activated by ECM molecules, matrix stiffness, and the degree of collagen crosslinking [1, 2, 14–16]. More recently, reprogramming of HSC metabolic program and epigenetic events have been identified as additional mechanisms driving HSC activation/deactivation program.

### 3.2. Hepatocytes

Hepatocyte apoptosis and/or necroapoptosis are key contributors of the fibrogenic process. Indeed, injured hepatocytes display enhanced oxidative stress, ER stress, and mitochondrial damage that are potent stimuli for hepatic stellate cell activation. Moreover, activated HSC display phagocytic properties towards hepatocyte-derived apoptotic bodies. Engulfment of apoptotic bodies results in enhanced resistance of HSC to apoptosis and increased profibrogenic properties [17] (Figure 1(b)).

### 3.3. Immune Cells

As described in other organs, sustained hepatic inflammation resulting from parenchymal liver injury is a major driving force of both fibrosis progression and fibrosis resolution, depending on cell type and activation state. Selective depletion of individual inflammatory cells has allowed characterizing the complex interactions and impact of innate (macrophages) and adaptive (T lymphocyte subsets) immune cells on fibrosis accumulation and regression.

#### 3.3.1. Innate Immune Cells

Activation of Kupffer cells and recruitment of monocyte/macrophages are a key event governing initiation, perpetuation, and resolution of fibrosis and has been extensively characterized, using pharmacological or conditional genetic ablation of monocytes/macrophages, in mice with ongoing liver injury [18, 19]. These studies have been corroborated by* in vitro* data showing that Kupffer cells promote activation and survival of HSC [19, 20]. However, macrophages harboring a distinct phenotype induce hepatic stellate cell apoptosis and produce active metalloproteinases that drive resolution of fibrosis [21]. Moreover, other innate immune cells have also been implicated. In particular, dendritic cells may orchestrate the inflammatory response during both progression and resolution of liver fibrosis [22–24]. NK cells reduce fibrogenesis by inducing apoptosis of early activated and senescent hepatic stellate cells via TRAIL [25, 26].

#### 3.3.2. Adaptive Immune Cells

CD4^+^ T lymphocytes (Th1, Th2, Th17, and Treg) control the fibrogenic process with positive or negative outcome depending on their phenotype [27]. Indeed, whereas Th1 effector T cells reduce liver fibrogenesis via the release of IFN-gamma, Th2 polarization promotes liver fibrosis via production of IL-13. T helper 17 (Th17) lymphocytes have also more recently emerged as critical enhancers of profibrogenic properties of hepatic myofibroblasts via secretion of IL17 [28–31]. The role of regulatory T cells has not been investigated as yet, but antifibrogenic properties might be anticipated from data obtained in cardiac and pulmonary fibrosis [27].

## 4. Autophagy and Liver Fibrosis

Currently recognized antifibrotic strategies include targeting of several steps leading to liver fibrogenesis, that is, inhibition of hepatocyte apoptosis, liver inflammation, and/or promotion of fibrogenic cell apoptosis or reversion of fibrogenic cell phenotype to a quiescent state. Autophagy has recently emerged as a novel but complex regulator of liver fibrosis, with profibrogenic effects relying on its direct contribution to the process of HSC activation but antifibrogenic properties via indirect hepatoprotective and anti-inflammatory properties.

### 4.1. Autophagy in Hepatic Stellate Cells: A Profibrogenic Process (Figure 1(a))

A number of cells maintain energy homeostasis through autophagic digestion of intracellular lipids (lipophagy); this process has been well characterized in hepatocytes [11].

Because the progressive loss of retinoid-containing lipid droplets is a feature of hepatic stellate cell activation, autophagy has been hypothesized to govern the activation process by digesting lipid droplets. Two groups recently independently reported that autophagy contributes to hepatic stellate cell activation* in vitro*, both in mice and human cells, and confirmed these findings in cells isolated from mice acutely exposed to either thioacetamide or carbon tetrachloride [32, 33]. These conclusions were drawn on the basis of an increase in LC3-II and a decrease in p62/SQSTM1 expressions upon hepatic stellate cell activation, associated with enhanced autophagic flux and the presence of a high number of autophagic vacuoles. Conversely, pharmacological inhibition of autophagy or downregulation by small interfering RNAs against* Atg5* or* Atg7* reduced the number of lipid droplets within HSC [32, 33]. The findings were also supported in mice and fibrotic liver samples from patients with hepatitis B, which showed increased levels of autophagy in HSC upon liver injury [32]. Further experiments allowed establishing a link between autophagy, elimination of lipid droplets, and myofibroblastic differentiation of HSC. Indeed, as previously shown in hepatocytes, autophagy enables catabolism of retinyl esters by lipases, thereby providing free fatty acids that increase generation of ATP following mitochondrial *β*-oxidation [32]. Potential signals triggering autophagy in hepatic stellate cell have been recently identified and they include oxidative stress and endoplasmic reticulum stress [34], which are recognized signals for hepatic stellate cell activation* in vitro* and in the injured liver. Altogether these data identify lipophagy of retinyl esters as a mandatory driver of the initiation and perpetuation of the activated phenotype of liver fibrogenic cells.

Another major finding of these studies was the demonstration of the profibrogenic consequences of autophagy activation in hepatic stellate cells. Indeed, when autophagy was blunted with pharmacological inhibitors or following genetic invalidation of the autophagic genes* Atg7* or* Atg5*, downregulation of the fibrogenic properties of cultured hepatic stellate cells was observed, as illustrated by HSC growth inhibition, reduced expression of fibrogenic genes and of the activation marker alpha SMA [32, 33]. These data were further confirmed* in vivo,* in mice harboring a specific deletion of the autophagic gene* Atg7* in hepatic stellate cells (*Atg*7^F/F^-GFAP-cre mice) that showed decreased hepatic stellate cell activation and reduced liver fibrogenesis and matrix accumulation upon chronic administration of carbon tetrachloride or thioacetamide [32]. These results demonstrated that autophagy in hepatic stellate cells contributes to the liver fibrogenic process. Importantly, inactivation of autophagy in kidney and lung fibrogenic cells also reduced their capacity to drive a fibrogenic response [32], identifying autophagy as a potential core pathway of fibrogenesis.

However, a more complex scheme is emerging, as recent data indicate that, in other hepatic cell types, autophagy reduces profibrogenic signals, by protecting hepatocytes from apoptosis [35] and eliciting anti-inflammatory effects in Kupffer cells [36].

### 4.2. Autophagy: A Protective Anti-Inflammatory Process with Antifibrogenic Properties (Figure 1(b))

Several studies have conclusively demonstrated the role of autophagy in the control of proinflammatory signaling [37]. In macrophages, autophagy regulates phagocytosis of pathogens and is critically involved in monocyte differentiation into macrophages and acquisition of phagocytic functions [38]. Interestingly, macrophages exposed to an autophagy inhibitor or lacking one of the autophagic components (Atg16L1, ATG5, ATG7, Beclin 1, or LC3B) display a proinflammatory phenotype, characterized by enhanced IL1*β* secretion, that results from ROS-mediated activation of the NRLP3 inflammasome pathway [39–41]. In addition, autophagy-defective (*Atg5*fl/fl* LysM*-Cre+) macrophages secrete high levels of IL1*α* through a ROS/calpain-dependent but inflammasome-independent pathway [42]. The central role of autophagic genes in the anti-inflammatory response of macrophages suggests that in the context of liver fibrosis, macrophage autophagy may be a protective pathway that prevents excessive release of inflammatory mediators during chronic liver injury. We recently addressed this hypothesis in mice lacking the autophagic gene* Atg5* in myeloid cells (*Atg5*fl/fl* LysM*-Cre+ mice) and uncovered the beneficial consequences of macrophage autophagy on liver fibrosis [36]. Indeed, these mice were more susceptible to liver inflammation and liver injury when exposed to carbon tetrachloride and showed higher hepatic secretion of IL1*α* and -*β*, increased recruitment of neutrophils and monocytes into the liver, and enhanced hepatocyte apoptosis. Administration of carbon tetrachloride to* Atg5*fl/fl* LysM*-Cre+ mice was also associated with exacerbated fibrosis accumulation in the liver and accumulation of fibrogenic cells [36]. In keeping with* in vivo* data, mechanistic studies confirmed the higher fibrogenic potential of hepatic myofibroblasts exposed to the conditioned medium of* Atg5*fl/fl* LysM*-Cre+ macrophages. This effect resulted from an increased release of IL1*α* and -*β* from* Atg5*fl/fl* LysM*-Cre macrophages, since it was blunted by IL1*α* and -*β* neutralizing antibodies. Overall, these data identify liver macrophage autophagy as an anti-inflammatory pathway, with protective antifibrogenic effects by paracrine interactions with hepatic myofibroblasts (Figure 1(b)) [36].

Autophagy also controls T cell activation, in part by regulating the inflammatory response of macrophages and dentritic cells. Thus, autophagy-deficient macrophages show excessive secretion of IL1*α* and IL1*β*, two cytokines that function together with IL-6 and TGF-*β* to promote Th17 differentiation and responses [42]; similarly, pharmacological inhibition of autophagy in dendritic cells enhances the production of inflammatory mediators from *γδ* T cells, including IL17 [43]. Concordantly, mice with selective deletion of autophagy protein in myeloid cells demonstrate enhanced inflammatory responses, including increased secretion/release of IL-1 and IL-17 in response to mycobacterium tuberculosis [42]. Given the reported profibrogenic role of IL-17 in the liver, whether macrophage and/or dentritic cell autophagy may also indirectly inhibit liver fibrosis via limitation of IL-17 release is an important issue that deserves further investigation.

### 4.3. Hepatoprotective Properties of Autophagy May Contribute to Inhibition of Fibrogenesis (Figure 1(c))

Although both survival and apoptotic properties of autophagy have been described, recent studies using more specific tools have established that autophagy is mainly a prosurvival pathway that removes misfolded proteins, accumulated lipids (lipophagy), and/or damaged mitochondria (mitophagy) to reduce oxidative stress and lipid peroxidation and supply nutrients to maintain cellular energy homeostasis under injured conditions.

In the liver, autophagy behaves as a protective pathway in the face of various forms of injury. Thus, in the context of nonalcoholic fatty liver disease, free fatty acids inhibit autophagy in hepatocytes, thereby inducing hepatocyte apoptosis; conversely, autophagy underlies resistance of hepatocytes to the apoptotic effects of free fatty acids [35]. Similarly, autophagy is a survival pathway against acute alcohol-induced hepatocyte apoptosis [44]. In response to acetaminophen intoxication, autophagy serves to remove damaged mitochondria (mitophagy) thereby providing regulatory loop protecting against hepatocyte necrosis [45]. Moreover, during ischemia/reperfusion injury suppression of autophagy triggers hepatocyte death [46]. Finally, in alpha-1 antitrypsin deficiency, pharmacological induction of autophagy limits the cellular aggregation of mutant *α*1-antitrypsin, thereby reducing liver fibrogenesis in rodents [47]. Interestingly, in this setting, autophagy induction provides protection towards both hepatocellular damage and liver fibrosis, in sharp contrast with the profibrogenic effects of autophagy in hepatic stellate cells. Moreover, we recently showed that* Atg5*fl/fl* LysM*-Cre+ mice with exacerbated liver fibrosis in response to CCl_4_ also display enhanced hepatocyte apoptosis [36], suggesting that macrophage autophagy might provide an additional hepatoprotective mechanism contributing to its antifibrogenic effects.

## 5. Autophagy in Liver Fibrosis: Friend or Foe?

**Figure 2:**
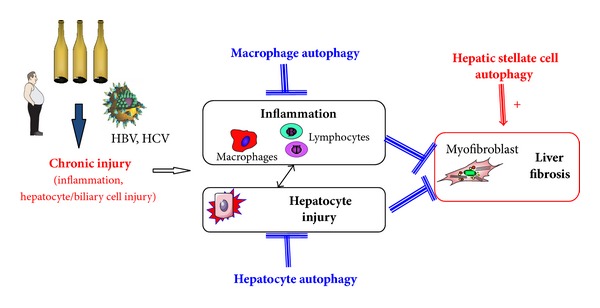
Autophagy in the liver: a pathway with divergent cell-specific effects? Autophagy enhances fibrogenic properties in hepatic stellate cells. In contrast, anti-inflammatory effects in macrophages and hepatoprotective effects in hepatocytes limit the development of liver fibrosis.

Autophagy has initially been viewed as a hepatoprotective and anti-inflammatory pathway during liver injury. However, a more complex paradigm is emerging with the identification of the profibrogenic effect of autophagy in fibrogenic cells (Figure 2). Therefore, because autophagy elicits divergent and cell-specific effects during chronic liver injury, manipulation of autophagy for therapeutic antifibrogenic purposes should only be considered by means of cell-specific delivery approaches.
